# Evaluation of Three Machine Learning Algorithms for the Automatic Classification of EMG Patterns in Gait Disorders

**DOI:** 10.3389/fneur.2021.666458

**Published:** 2021-05-21

**Authors:** Christopher Fricke, Jalal Alizadeh, Nahrin Zakhary, Timo B. Woost, Martin Bogdan, Joseph Classen

**Affiliations:** ^1^Department of Neurology, University Hospital of Leipzig, Leipzig, Germany; ^2^Faculty of Mathematics and Computer Science, Leipzig University, Leipzig, Germany; ^3^Department of Psychiatry and Psychotherapy, Center for Psychosocial Medicine, University Medical Center Hamburg-Eppendorf (UKE), Hamburg, Germany

**Keywords:** machine learning, gait disorder classification, convolutional neural network, support vector machine, k nearest neighbor

## Abstract

Gait disorders are common in neurodegenerative diseases and distinguishing between seemingly similar kinematic patterns associated with different pathological entities is a challenge even for the experienced clinician. Ultimately, muscle activity underlies the generation of kinematic patterns. Therefore, one possible way to address this problem may be to differentiate gait disorders by analyzing intrinsic features of muscle activations patterns. Here, we examined whether it is possible to differentiate electromyography (EMG) gait patterns of healthy subjects and patients with different gait disorders using machine learning techniques. Nineteen healthy volunteers (9 male, 10 female, age 28.2 ± 6.2 years) and 18 patients with gait disorders (10 male, 8 female, age 66.2 ± 14.7 years) resulting from different neurological diseases walked down a hallway 10 times at a convenient pace while their muscle activity was recorded via surface EMG electrodes attached to 5 muscles of each leg (10 channels in total). Gait disorders were classified as predominantly hypokinetic (*n* = 12) or ataxic (*n* = 6) gait by two experienced raters based on video recordings. Three different classification methods (Convolutional Neural Network—CNN, Support Vector Machine—SVM, K-Nearest Neighbors—KNN) were used to automatically classify EMG patterns according to the underlying gait disorder and differentiate patients and healthy participants. Using a leave-one-out approach for training and evaluating the classifiers, the automatic classification of normal and abnormal EMG patterns during gait (2 classes: “healthy” and “patient”) was possible with a high degree of accuracy using CNN (accuracy 91.9%), but not SVM (accuracy 67.6%) or KNN (accuracy 48.7%). For classification of hypokinetic vs. ataxic vs. normal gait (3 classes) best results were again obtained for CNN (accuracy 83.8%) while SVM and KNN performed worse (accuracy SVM 51.4%, KNN 32.4%). These results suggest that machine learning methods are useful for distinguishing individuals with gait disorders from healthy controls and may help classification with respect to the underlying disorder even when classifiers are trained on comparably small cohorts. In our study, CNN achieved higher accuracy than SVM and KNN and may constitute a promising method for further investigation.

## Introduction

Gait disorders are a common accompaniment of many neurological diseases (1). They represent a major health hazard as they are a frequent cause of falls, with consecutive morbidity and mortality, and they reduce the quality of life of affected patients by impairing their ability to perform activities of daily living and to participate in normal social life (1, 2). In addition, they cause considerable economic costs in the healthcare sector (3–5).

A wide variety of pathological entities affecting different structures of the central or peripheral nervous system may underlie gait disorders. Involvement of any part of the nervous system is initially usually derived from the neurological examination. However, due to their phenotypical similarity, classification of diseases with prominent neurological gait disorders is only moderately accurate even for more experienced clinicians. Since the accurate identification of the underlying aetiopathogenesis of a gait disorder is important for planning its diagnostic workup, treatment, and prognosis, a more precise classification of gait disorders would probably improve the quality of medical care and avoid unnecessary and costly diagnostic or therapeutic procedures. In order to analyze gait traits, kinematic recordings and surface electromyography (EMG) signals have been used previously in a number of studies. Although analysis of kinematic data may be more intuitive, EMG signals may have the advantage of being nearer to the neuronal control mechanisms active during normal and pathological walking (6–8).

In this study, we aimed to investigate the hypothesis that muscle activation patterns during walking may contain enough information to allow a classification into classes of gait disorders, sufficiently accurate to help improve the classification by clinical assessment, which has been shown to be in the order of 50 to 80% (9–12). Due to the high dimensionality and complexity of multichannel EMG data and the uncertainty with respect to the features determining pathological or healthy gait, we employed data-driven technical approaches which rely on non-linear classifiers. We specifically asked whether machine learning techniques applied to EMG signals would be able to (i) accurately separate patients from healthy controls and (ii) separate two major classes of gait impairments commonly found in neurology (hypokinetic and ataxic gait) from each other and from healthy subjects. In addition, our study served to explore the question of whether machine learning methods can be meaningfully applied to small datasets.

For automatic classification multiple approaches are available. A major advantage of neural networks may be their ability to differentiate non-linear relationships between data which are not easily tractable by linear algorithms such as multiple linear regression (13). Since different classification algorithms perform differently depending on the properties of the dataset to be classified, we used three different algorithms that have been used previously successfully for the classification of kinematic and/or EMG gait data: Convolutional Neural Network (CNN), Support Vector Machine (SVM) and K-Nearest Neighbors (KNN) (14–19).

## Materials and Methods

The protocol was approved by the local Ethics Committee (no. 271-15-13072015) and all procedures were performed according to the Declaration of Helsinki. Written informed consent was obtained from all participants.

### Participants

Patients with different neurological diseases were included (Results section, Table 1). Inclusion criteria for study participation were age of 18 years or above and presence of a neurological gait disorder falling into the categories “hypokinetic gait” and “ataxic gait” for patients and the absence of any gait disorder for healthy subjects. Exclusion criteria were inability to walk freely, severe orthopedic, neuropsychiatric or medical disorders interfering with safe participation as well as present pregnancy. Healthy controls were recruited using public displays and from medical staff not involved in conducting the study.

**Table 1:** Patient characteristics.

**Age range (years)**	**Gender**	**Disease**	**Timed-up-and-go test (seconds)**	**MDS-UPDRS-III gait sub-score**	**SARA gait sub-score**	**Classified as**
70–80	M	PD	13.7	3	2	Hypokinetic
50–60	F	PD	5.8	1	0	Hypokinetic
60–70	M	PD	7.7	6	1	Hypokinetic
50–60	F	PD	7.7	1	0	Hypokinetic
70–80	M	PD	15.6	3	1	Hypokinetic
70–80	F	PD	10.1	5	2	Hypokinetic
50–60	M	PD	9.1	2	0	Hypokinetic
70–80	F	PD	17.4	3	1	Hypokinetic
70–80	M	PD	17.5	4	2	Hypokinetic
70–80	M	PD	53.5	7	1	Hypokinetic
60–70	M	CA	15.2	3	3	Ataxic
60–70	F	CA	16.0	4	4	Ataxic
70–80	M	CA	15.2	1	3	Ataxic
70–80	F	CA	25.1	2	2	Ataxic
20–30	F	MS	11.8	0	1	Ataxic
30–40	F	MS	8.5	1	3	Ataxic
70–80	M	MSA	14.8	4	1	Hypokinetic
70–80	M	NPH	25.2	8	3	Hypokinetic

*M, male; F, female; PD, Parkinson's Disease; CA, cerebellar ataxia; MS, multiple sclerosis; MSA, multiple system atrophy; NPH, normal pressure hydrocephalus*.

### Experimental Procedures

Prior to the main experiment, all participants performed a Timed-up-and-go test (20). Afterwards, they were instructed to walk down a hallway at a self-selected pace, then to turn and perform a tandem gait. Participants were filmed during the gait task. These tasks allowed us to visually detect features that are used to clinically classify patients into the two categories of gait disorders we wanted to investigate: hypokinetic gait and ataxic gait. Clinical classification was done offline by inspection of the videos by two experienced raters (C.F., T.B.W.). Part III of the Movement Disorder Society Unified Parkinson's Disease Rating Scale [MDS-UPDRS-III (21)] is commonly used to assess symptom severity in Parkinson's Disease and items 3.10 and 3.11 represent hypokinetic gait impairment. We determined the value of both items from the videos and summed them to generate a MDS-UPDRS-III sub-score. The Scale for the Assessment and Rating of Ataxia [SARA (22)] is a clinical score for ataxia patients. It also features a gait disorder item for ataxic gait impairment (item 1) which also was derived from the videos.

For the main experiment 10 bipolar surface EMG electrodes (Noraxon Dual EMG Electrodes, Noraxon, Scottsdale, USA) recorded in differential derivation were attached to five muscles of each leg: rectus femoris, vastus medialis, biceps femoris, tibialis anterior, gastrocnemius lateralis muscle. Additionally, a tri-axial accelerometer was attached to each foot whose data was used to detect the beginning and end of each gait cycle. EMG and accelerometer data were recorded using a wireless EMG system (DTS Desk Receiver, Noraxon, Scottsdale, USA) and sampled at 1,500 Hz. Subjects were instructed to sit down, stand up, then walk down a corridor for a distance of 8 meters, turn around a pole, walk back and sit down again. This procedure was repeated 10 times. They were filmed during the procedure which allowed selecting EMG data belonging to epochs where subjects were freely walking. The placement of electrodes was carried out by the same experimenter (N.Z.) for each participant. Electrode positions were determined using known anatomical landmarks for the target muscles.

### Data Pre-processing

All EMG and accelerometer datasets were exported to Matlab (MathWorks, Natick, USA) and analyzed offline using custom software written in Matlab and Python (Python Software Foundation, Delaware, USA). Videos of the walking experiment were epoched to contain only walking, removing epochs where participants either were sitting, standing or turning.

For data pre-processing, a zero-lag 5th order Butterworth high-pass digital filter with 20 Hz cut-off frequency was applied to remove DC components and low frequency noise due to movement and varying electrode-skin contact. Afterwards, we applied a zero-lag 5th order low-pass Butterworth filter with a cut-off frequency of 400 Hz to the data in order to reduce higher frequency artifacts and performed full-wave rectification.

Based on the accelerometer data all EMG data was further epoched such that each epoch represented a single gait cycle (stance and swing phase for each leg, corresponding to a consecutive step with each leg). The gait cycle was determined using the recordings from the accelerometers places on both feet. Because the accelerometers were mounted on the feet a large signal was recorded from an accelerometer each time when the foot onto which it was mounted hits the ground during walking. In detail, the 3 channels of each tri-axial accelerometer placed on each foot were merged and the absolute values were calculated according to:

$$\begin{array}{l} x_{m}\left(t\right)=\sqrt[{}]{x_{1}{\left(t\right)}^{2}+x_{2}{\left(t\right)}^{2}+x_{3}{\left(t\right)}^{\text{2}}} \end{array}$$

*x*_m_*(t)*… merged accelerometer data at time *t, x*_1_*(t)* to *x*_3_*(t)* … individual channel data at time *t*.

**Figure 1:**
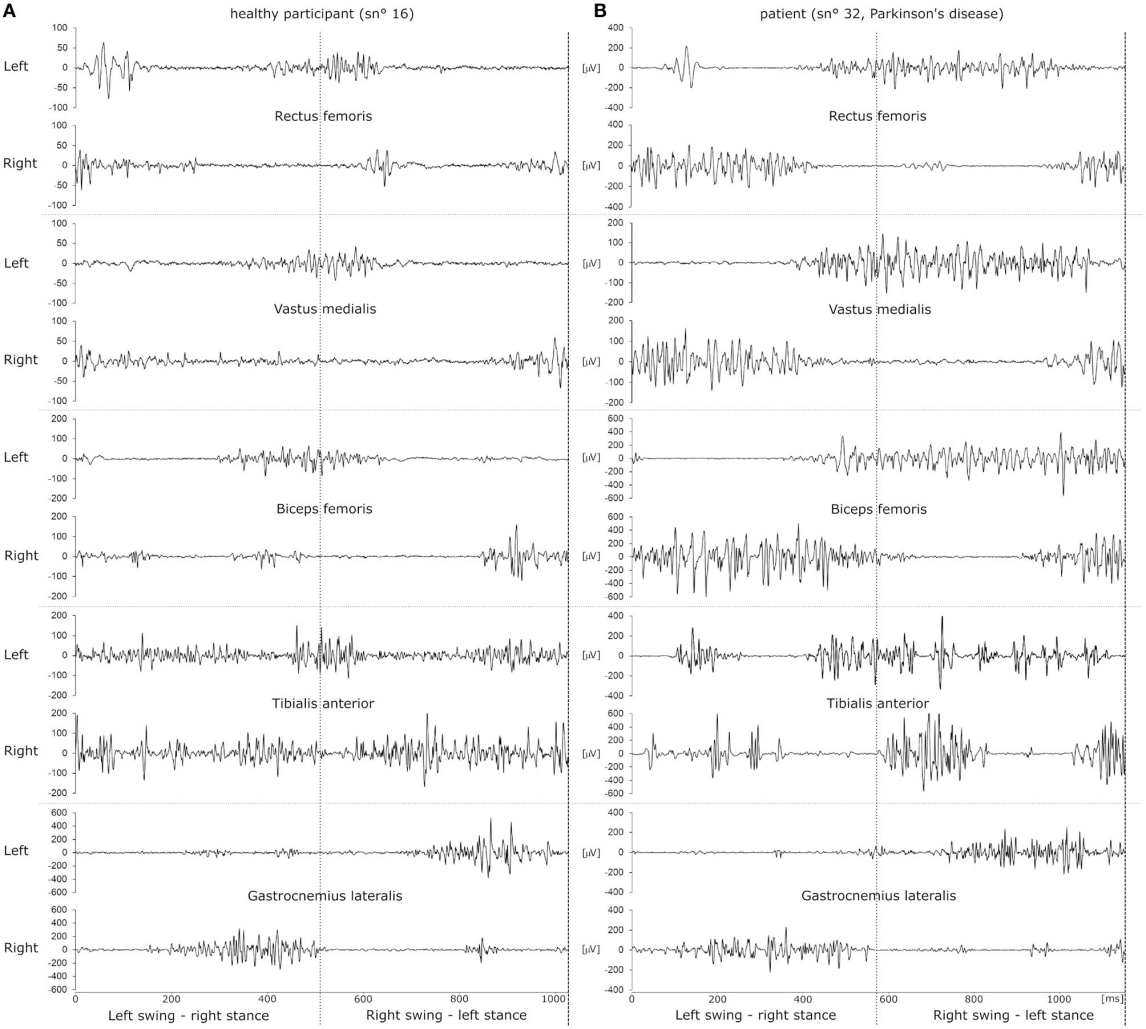
Example raw EMG datasets of a single gait cycle of a healthy participant **(A)** and a patient with Parkinson's disease **(B)**. EMG traces recorded from left and right leg muscles are depicted as pairs above one another. The dotted vertical line in the middle of the panels illustrates the time of ground contact with the left foot. Ground contact with the right foot happens at 0 ms and at the end of the dataset, indicated by a dashed line. Thus, the left leg transitions into swing phase in the first half of the dataset while the right foot is placed on the ground and vice versa for the second half of the dataset.

*x*_m_ was then normalized and a composite single channel was computed by subtracting *x*_m_ of the right side from *x*_m_ of the left side. Matlab *envelope* was used to obtain clear smooth peaks, which were extracted with Matlab *findpeaks*. A ground impact with the right foot yielded a strong negative peak immediately followed by a positive peak when the swing phase of the left leg happened while the right foot rested on the floor. Using this, a gait cycle was thus defined based on two consecutive impacts with the right foot. Examples of EMG signals sampled during a gait cycle are depicted in Figure 1.

Due to inter- and intra-individual variability each gait cycle was of different duration and, therefore, dataset epochs were of different length. To enable comparison within and across subjects we resampled the data linearly to fix all datasets to a common length (1,500 datapoints, Matlab *resample* using a FIR antialiasing low pass filter) across all muscles and subjects.

### Classification Algorithms

We employed three supervised classification algorithms to automatically classify gait data according to the algorithm-based and manually extracted features which are described in the following subsection: Convolutional Neural Network (CNN), Support Vector Machine (SVM) and K-Nearest Neighbors (KNN). The algorithms were supposed to classify (1) healthy vs. gait disorder and (2) healthy vs. hypokinetic vs. ataxic gait.

#### Convolutional Neural Network (CNN) Classifier

A CNN is a type of deep neural network classifier which historically had applications in image classification and recognition (23, 24). The main advantage of a CNN classifier lies in its ability to recognize features more or less directly from the data instead of manually extracted features.

**Figure 2:**
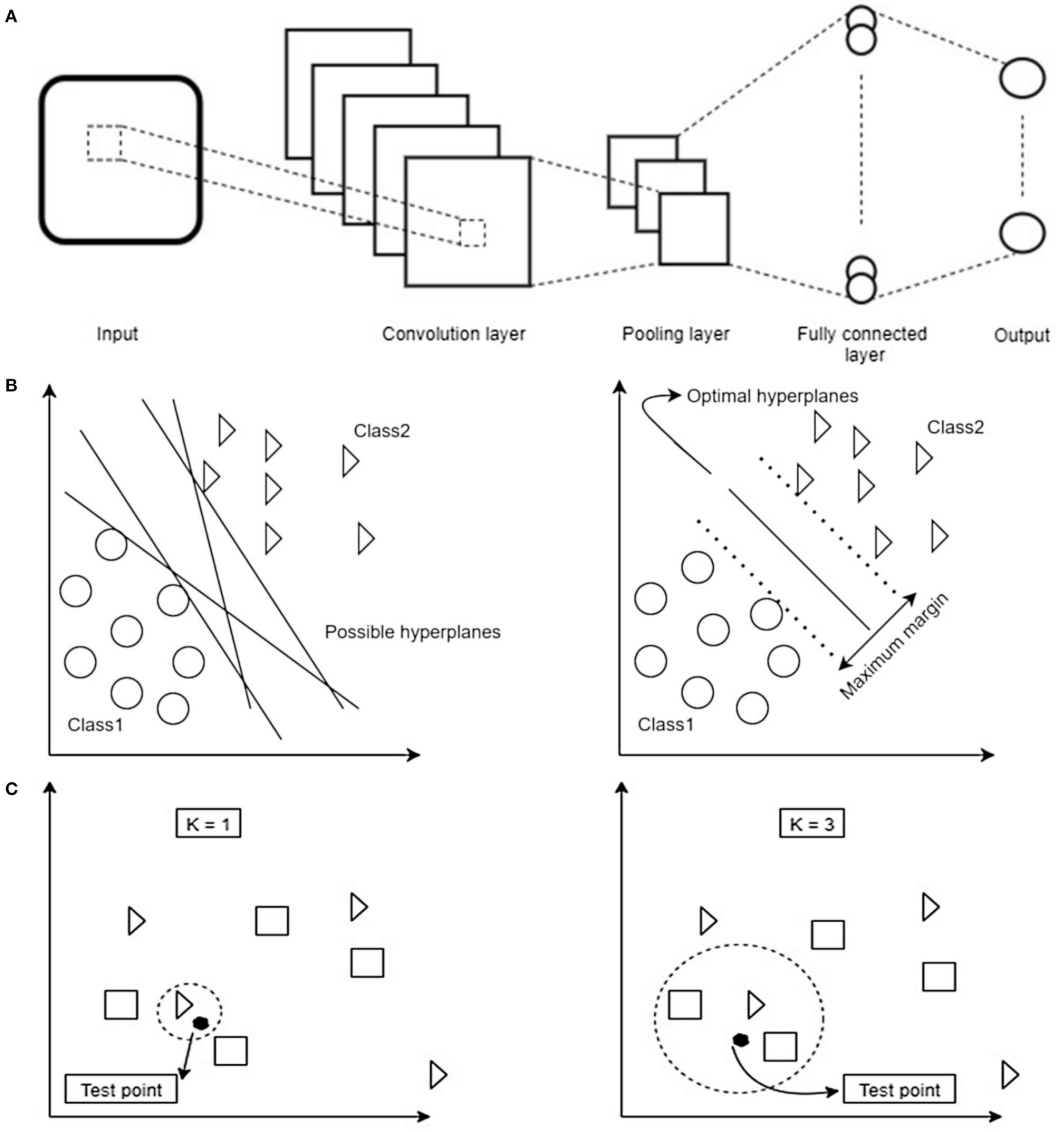
**(A)** Schematic overview of the dataflow in a Convolutional Neural Network (CNN). Image data is fed into the network as an input. In order to reduce the data dimensionality, different convolution and pooling layers are used. Finally, to have the classification, fully connected layer is used to flatten the output of the previous layer. **(B)** Schematic overview of how a Support Vector Machine (SVM) selects the best hyperplane for classification. First hyperplanes are calculated which are able to optimally separate data points. From all possible hyperplanes, the algorithm chooses the optimal one maximizing the distance between the hyperplane and the points of two classes. Note that in 2D the hyperplane corresponds to a line. Based on the kernel function chosen this can have different shapes. **(C)** Schematic overview of decision boundaries in a K-Nearest Neighbors (KNN). Distance between test- and training data points are calculated. Afterwards, based on the value of K, the algorithm decides which class a given test point belongs to (in the example being a triangle or a square). Depending on the value of K different classification results are possible: on the left the test point would be classified as a triangle, on the right as a square.

Within the CNN data is put through a number of layers with different tasks (Figure 2A). The input is first fed into the convolution layer where a spatial filter in form of a window of weights is applied to the inputs. This spatial filter is moved vertically and horizontally throughout the input. Afterwards, the output of the convolution layer is rectified by a Rectified Linear Unit (ReLU) and then fed into the pooling layer, which reduces the dimensionality of the previous layer while preserving the spatial invariants. Depending on the number of convolution and pooling layers, different feature maps can be obtained that can affect the classification results (25). The results of the pooling layer are put through a fully connected layer yielding the classification.

Prior to training of the CNN, the datasets were converted to the time-frequency domain using continuous wavelet transform (26) in order to enhance the classification results, as previous studies have shown that the representation of time series data in the time-frequency domain has some advantages resulting in a more robust classifier (27, 28).

One of the key issues with implementing the CNN on a small-size dataset is the choice of filters, weights and number of layers to achieve a good performance while avoiding overfitting as these parameters play a major role in the calculation of the loss function (29). To overcome these problems, transfer learning can be a promising approach. This is based on the idea that one needs to select relevant parts of a pre-trained CNN which then are fine-tuned further for the problem at hand. Work by Pan and Yung (30) showed that transfer learning techniques can be employed even if two datasets are from different domains (30). This approach may be of particular interest in medical applications where datasets are often of limited size. This has been successfully demonstrated in recognition tasks in medical images where pre-trained CNNs generated on larger datasets provided a solution to dealing with small data samples (31). Since our sample was small, we applied this transfer learning method to our problem. In particular, we used the AlexNet model, which is a CNN pre-trained network on 1.2 million images of 22,000 categories (e.g., cats, dogs etc.) into 1,000 different classes using 25 layers (23, 32). From the pre-trained AlexNet model we used the first 22 layers (31) and added one fully-connected layer which was trained with our data. In addition, two Softmax and the classification output layers were added. During the training phase, an epoch was defined as a full training cycle using the whole training set and a mini-batch size was a subset of the training set used to update the weights and calculate the gradient descent loss function. The training was completed after 10 epochs with a mini-batch size of 10 images when the learning rate was set to 0.0001.

#### Support Vector Machine (SVM) and K-Nearest Neighbors (KNN) Classifier

SVM (33) and KNN (34) constitute linear and non-linear classifiers which use different, and possibly high dimensional, features of the datasets to assign a label to a data point based on their location in the data space. SVM as a supervised learning algorithm has many applications, e.g., in face detection (35) and bioinformatics (36). It aims to find an optimal hyperplane in an N-dimensional space. This hyperplane is used as a decision boundary separating datasets into two or more classes without computing in the hyperplane due to the kernel trick (37). Thereby, it aims to maximize the distances of the data points of all classes to the hyperplane finding the so-called maximum margin hyperplane (Figure 2B). KNN (K-Nearest Neighbors) is another example of a supervised classification algorithm which has no explicit training phase (34). This algorithm is mainly applicable when there is little or no pre-knowledge about the relationship of the data points. In general, the KNN algorithm tries to classify a new instance based on the similar objects from the training set (38). KNN classification is based on a distance metric which is calculated between a data point to be classified and already known data points from a training set. In particular, the classifier assigns that label to a data point which is shared by the majority of the K nearest surrounding data points of the training set (Figure 2C). The classification result, therefore, not only depends on the distribution of data points in the parameter space but also on the value of K.

To enable SVM and KNN classification a number of features have been extracted from the datasets. In our study we extracted 7 features from each EMG channel according to previously published work (39, 40). Of these, 4 were calculated in the time-domain and 3 in the frequency-domain after Fast-Fourier-transformation (time-domain features: integrated EMG (IEMG), simple square integral (SSI), variance (VAR), root mean square (RMS), frequency-domain features: area of power, spectral moment SM1 and peak frequency (PKF). The computation of each feature is reported in Supplementary Table 2. Each feature was calculated and normalized per muscle resulting in a total of 70 features per subject (7 features x 10 muscles). Subsequently, each feature distribution was standardized to a mean of 0 and standard deviation of 1 in order to avoid range effects on the learning algorithm. The full number of 70 features appeared to be too large for training the classifiers without risking overfitting. Furthermore, multiple features for different muscles possibly represent similar aspects of the captured movement thus rendering them redundant. To account for these considerations we used principal component analysis (PCA) and selected as many principal components as to explain 99% of data variance (41) to feed the classifiers.

We employed a grid search approach to find optimal SVM and KNN hyperparameters. We tested the following values as hyperparameters for SVM: C = {0.01,0.03,0.1,0.3,1,3,10,30}, Gamma = {0.01,0.03,0.1,0.3,1,3,10,30} and for KNN: K = {1,2,3,…,30}.

### Training of the Classifiers, Classifier Performance, and Statistical Procedures

After pre-processing, we randomly divided the datasets into training and validation sets in which the validation sets consisted of gait cycle EMG data of 14 out of 37 subjects with all their trials, while the training set consisted of the data of 22 participants, leaving data from one participant out of both the training and the validation set. After training (CNN, SVM) or setting up (KNN) the classifiers the remaining left-out subject was then used as the test set. Hyperparameters for KNN and SVM were also optimized using only the training and validation datasets, leaving out the test data, the hyperparameters are reported in Supplementary Table 2. For the CNN, the parameters and setup were kept identical. This procedure was repeated for all 37 subjects. In one analysis the left-out subject was classified as belonging to one of 2 classes (“healthy” or “patient”) and in the other analysis the left-out subject was classified as belonging to one of 3 classes (“healthy,” “hypokinetic,” or “ataxic”). The classification was done for each gait cycle trial and the classification result for the subject was defined as the label which was obtained most often when all gait cycles were considered. Importantly, due to the leave-one-out approach, the classifiers were trained without ever “seeing” any trials of the subject to be classified in the training phase. The leave-one-out procedure was then repeated by cycling through each participant as the left-out subject. Finally, we evaluated the classification result by calculating sensitivity, specificity and accuracy, for the 2-class problem according to:

$$\begin{array}{l} accuracy=\;\frac{TP+TN}{total\;number}\;sensitivity=\frac{TP}{TP+FN}\\ \;specificity=\;\frac{TN}{FP+TN} \end{array}$$

*TP … true positives, TN … true negatives, FP … false positives, FN … false negatives*.

For the 3-class problem we used the following formulae:

$$\begin{array}{l} accuracy=\;\frac{TPclass}{totalnumber}\;sensitivity\;\left(hypo/atax\right)=\frac{TP}{TP+FN}\\ \;specificity\;\left(hypo/atax\right)=\;\frac{TN}{FP+TN} \end{array}$$

*TP class … sum of true positive number of each class, TP … true positives for the class of interest, TN … true negatives for the class of interest, FP … false positives for the class of interest, FN … false negatives for the class of interest*.

Note, that in the 3-class problem the accuracy is calculated as a global metric while sensitivity and specificity are calculated for each of the pathological traits.

We employed chi-squared tests on the contingency tables to analyse whether classification results demonstrated significant deviation from a random classifier as well as *t*-tests (after confirming normal distribution using the Jarque-Bera test) to compare patient characteristics.

In order to evaluate whether the trained classifiers performed significantly better than random classifiers, we computed chance levels for the 2-class and 3-class problem based on the amount of trials. For an infinite number of trials the chance levels for a given classification label are at 50% for a 2-class and 33.33% for a 3-class classification problem. However, Combrisson and Jerbi (42) demonstrated that the chance level for a smaller dataset depends on the number of trials. We therefore applied a correction when reporting whether classifier accuracies surpass chance level as described previously (42, 43):

$$\begin{array}{l} Std\left(\alpha \right)=binoinv\left(1-\;\alpha ,\;n,\;\frac{1}{c}\right)\times \frac{100}{n} \end{array}$$

α…* type I error level* = *0.05, n … number of trials corresponding to each subject or number of subjects, c … number of classes*.

The amount to which chance levels were surpassed were compared between the 3 classifiers using the Kruskal-Wallis test. When results were found to be significant we employed Mann-Whitney-U tests as *post-hoc* tests with Bonferroni correction where appropriate. Significance was defined at an alpha threshold of 0.05.

Chance levels were also calculated based on the number for individual gait cycles. When a classifier assigns a given label to a fraction of gait cycles of a participant this can be considered as a measure of confidence or reliability with which a certain label is assigned by a classifier.

## Results

### Patient Characteristics

Nineteen healthy subjects (9 male, 10 female, age 28.2 ± 6.2 years) and 18 patients with gait disorders (10 male, 8 female, age 66.2 ± 14.7 years) resulting from different neurological diseases participated in our study (Table 1). Patients suffered from Parkinson's Disease (10), cerebellar ataxia (4), multiple sclerosis (2), multiple system atrophy (1) and normal pressure hydrocephalus (1). Based on clinical examination, gait was considered abnormal in all patients, reflecting the inclusion criterion. Gait disorders were differentiated into hypokinetic (12) or ataxic (6) gait based on their clinical presentation. SARA sub-scores for patients were significantly higher for ataxia patients compared to hypokinetic patients (two-sample *t*-test, *p* = 0.014). MDS-UPDRS-III gait sub-scores on the other hand were higher for hypokinetic patients (two-sample *t*-test, *p* = 0.033). The Timed-up-and-go test was performed significantly faster by healthy participants compared to patients (healthy: 7.3 ± 1.1 s, patients: 16.5 ± 10.6 s, two-sample *t*-test, *p* < 0.001).

### Classification Results

We trained CNN, SVM and KNN classifiers on our EMG data to discriminate between normal and pathological gait patterns. CNN was able to correctly classify the EMG datasets with a sensitivity of 94.4% and a specificity of 89.5% yielding an accuracy of 91.9% (chi-squared test, *p* < 0.001, Table 2). KNN classification yielded a sensitivity of 83.3% and a specificity of 15.8% resulting in an accuracy of 48.7% (chi-squared test, *p* = 0.943). SVM performed in between the other two classifiers with a sensitivity of 83.3%, a specificity of 52.6% and an accuracy of 67.6% (chi-squared test, *p* = 0.022).

**Table 2:** Contingency table for CNN classification in 2 classes.

	**Classification as “healthy”**	**Classification as “patient”**	**Σ**
Healthy group	17	2	19
Patient group	1	17	18
Σ	18	19	37

As a measure of classifier reliability, chance levels for individual subject step classification were calculated and found to be 54.5 ± 2.9% for CNN and 64.8 ± 11.0% for SVM and KNN. Classification using CNN surpassed these individual chance levels on average by 20.7 ± 14.5%, KNN by 0.8 ± 17.6%, and SVM by 9.0 ± 2.1% (Kruskal-Wallis test, *p* < 0.001). *Post-hoc* tests were significant for CNN compared to KNN (*p* < 0.001) and SVM (*p* = 0.005). Using CNN the frequency of an assigned label to the steps of participants was found to be above chance level for 86.5%, with KNN for 56.8%, and with SVM for 59.5% of participants, indicating the best reliability of the CNN.

All three algorithms were trained again to classify EMG patterns with respect to healthy, hypokinetic and ataxic gait. We found that CNN reached an accuracy of 83.8% with a sensitivity of 91.7% and a specificity of 84.0% for hypokinetic gait, and a sensitivity of 50.0% and a specificity of 93.6% for ataxic gait. The mean sensitivity was 70.8%, mean specificity 88.8% (contingency table shown in Table 3, chi-squared test, *p* < 0.001). The KNN model achieved a sensitivity of 25.0% with a specificity of 66.7% for hypokinetic gait and a sensitivity of 64.0% with a specificity of 77.4% for ataxic gait. The mean sensitivity was 45.8%, mean specificity 70.7% and accuracy was 32.4% (chi-squared test, *p* = 0.061). For SVM we computed a sensitivity of 33.3% and a specificity of 72.0% for hypokinetic gait as well as a sensitivity of 16.7% and a specificity of 93.6% for ataxic gait. The accuracy was 51.4% (chi-squared test, *p* = 0.086). The mean sensitivity was 25.0%, mean specificity was 82.8 %.

**Table 3:** Contingency table for CNN classification in 3 classes.

	**Classification as “healthy”**	**Classification as “hypokinetic gait”**	**Classification as “ataxic gait”**	**Σ**
Healthy group	17	1	1	19
Hypokinetic group	0	11	1	12
Ataxic group	0	3	3	6
Σ	17	15	5	37

Regarding classifier reliability, chance levels for individual subject step classification were computed to be 37.7 ± 2.7% for CNN and 47.5 ± 9.4% for KNN and SVM. Classification employing CNN surpassed these levels on average by 28.6 ± 15.5%, KNN by 1.7 ± 11.7%, and SVM by 20.9 ± 17.4% (Kruskal-Wallis test, *p* = 0.001). *Post-hoc* tests were significant for CNN compared to KNN (*p* < 0.001) and SVM compared to KNN (*p* < 0.001). The frequency of assigned labels to steps were above chance level for 97.3% of participants with CNN, 51.4% with KNN, and 91.9% with SVM, thus reliability was high for CNN and SVM.

For SVM and KNN hyperparameters (C and gamma for SVM, K for KNN) which were found to be optimal during grid search were widely distributed throughout the whole tested range (Supplementary Table 2).

In summary, in this application scenario CNN performed best in classifying EMG gait cycle patterns—using different pre-processing steps and directly recognizing features—in comparison to the other classifiers yielding best sensitivity, specificity and accuracy for the 2-class and 3-class problems. CNN and SVM classified reliably above chance level but CNN showed a markedly higher accuracy. Also SVM tended to reliably but erroneously classify a given subject to a label in the 3-class problem, suggesting that SVM is following a certain intra-individual but not an interclass pattern. Finally, the strong variability of optimal hyperparameters for SVM and KNN suggests a poor generalizability of both models while CNN architecture was left constant throughout the study.

## Discussion

We examined the ability of three different classifiers to automatically classify EMG data recorded during walking with respect to whether datasets were recorded from healthy subjects or patients with gait disorders as well as to classify the type of gait disorder. We found that classifiers based on a Convolutional Neural Network (CNN) worked best while Support Vector Machine (SVM) and K-Nearest Neighbors (KNN) classifiers performed considerably poorer. Results were consistent and a classification accuracy for an unknown dataset in the order of 80% or better was achieved while clinical diagnoses of movement disorders associated with gait disorders reach an accuracy of 50 to 80% (9–12).

There have been a multitude of studies investigating physiological and pathological aspects of human walking with a large number of different analytical techniques applied to either kinematic or EMG data (13, 44). Due to some technical advantages, the use of kinematic data in gait analysis is often favored, as EMG data contains a large proportion of inter-individual variance (45) and is subject to noise and electrical artifacts. A large number of possible and interdependent parameters can be extracted from both kinematic and EMG data. Under certain circumstances a single parameter or a limited set of parameters may reliably differentiate between different pathological conditions (46, 47). However, it is likely that different sets of parameters are important for different disorders. Therefore, analytical approaches must consider a larger number of parameters and their non-linear codependences if they are to differentiate between multiple pathological conditions. A potential advantage of using EMG data may result from the technique being more proximal to the actual neural control of movement than kinematic data and, therefore, it may contain additional information on the neurobiological underpinnings resulting in different gait traits and disorders important for classification. To obtain information on underlying muscular activity patterns during walking matrix factorization techniques like PCA, factor analysis and non-negative matrix factorization have been used to reduce data dimensionality while retaining data variance. Using this approach, a small number of activation patterns of synergistically activated muscles have been identified during walking (7, 8, 45, 48). Evidence revealing the presence of EMG patterns in walking behavior suggests that EMG data may contain enough information to drive an automatic classifier for differentiating healthy and impaired gait, as neuronal control mechanisms most likely are measurably altered in gait disorders.

In order to achieve a useful classification we investigated three machine learning techniques of which CNN proved to be most feasible to classify EMG signals based on multidimensional hidden features. In previous studies, similar approaches (SVM, Random Forest analysis and CNN classifiers) have been used successfully mostly on kinematic data to identify gait traits (15, 18, 49–51). With respect to the automatic classification of gait disorders a comparably small number of studies demonstrated promising results. Of note, Pradhan and colleagues (52) extracted a number of kinematic parameters associated with walking patterns from sensors imbedded in a carpet (GAITRite sensor carpet) in a larger study with the aim to classify different gait disorders. The classification was done in 120 patients suffering from 4 types of diseases (phobic postural vertigo, cerebellar ataxia, progressive supranuclear palsy, bilateral vestibulopathy) and 30 controls, the accuracy for the automatic classification amounted to some 90%, while our present approach yielded an accuracy of 80 to 90 %.

With respect to automatic gait disorder classification with EMG signals, only a limited number of observations have been published which also showed the feasibility of the approach. In orthopedic gait disorders a recent study successfully distinguished patients with and without knee injuries based on EMG data using a SVM approach (19). A similar concept was employed by Nair and colleagues who also found that automatic classification was feasible in a comparably small dataset with 18 patients (53). In both studies a similar classification accuracy was found around 80 to 90%. Although the analytical strategy was similar to our study, an important difference lies in the origin of altered motor activity in orthopedic disorders. In these disorders alterations of motor activity likely reflect adaptive or alternative movement strategies secondary to peripheral abnormalities whereas gait abnormalities in the present study, by design, principally originated in a central nervous system disorder. This may also be the reason why SVM was suitable in the studies by Mohr and colleagues (19) and Nair and colleagues (53), but not in our study. It appears to suggest that classifiers need to be carefully evaluated in the context in which they are applied. Nevertheless, although we discussed that EMG might be superior to kinematic data in classifying gait disorders, this conjecture must remain speculative, as we are not able to directly compare classification results based on both approaches given our current data.

Albeit non-linear classifiers have been shown to be useful in a variety of classification tasks, the way the trained classifiers achieve the output results remains hidden, even though new techniques evolve which may allow identification of the underlying processes driving the classification (54). The “black box” of a deep learning artificial neural network is notoriously difficult to interpret from neurobiologically as non-linearly weighted combinations of single or multiple parameters in various combinations drive the classifications. Also there are a number of pitfalls which need to be taken into account. Probably the most pertinent problem is overfitting of parameters which refers to the phenomenon that a classifier learns statistical features which are specific for the training set but might not be generally present (e.g., certain noise), but may still result in excellent classification results (29). This is of particular concern in studies involving small cohorts such as the present one and in datasets where the impact of individual samples and accidental associations is large. Since each individual performs a number of trials which are taken together and split up randomly into training, validation and test sets, data from each participant will basically be present in all sets, resulting in a good classification result during testing because the classifier was already trained with data very similar to the test set. This may occur despite the fact that the classifier has not learned biologically important discriminant data features. To guard against this problem we, first, reduced the number of parameters with PCA (41). Secondly, we employed a leave-one-out approach by testing the classifier only on data which was not used to train the classifier. These two measures were designed to avoid that the classifiers were trained on the noisy aspects of the dataset instead of the underlying data structure.

The reason for the different performances of the classifiers most likely is a result of inherent properties of the non-linear classifiers in light of limited dataset sizes. Though KNN and SVM are known to reliably work in smaller datasets, as has been shown in a number of the studies discussed above (15, 18, 19, 53), they may not be suitable for classification in this case because of complex interactions of differences which cannot be captured by hyperplanes (SVM) and even worse by spatial proximity (KNN). On the other hand, CNN may avoid these methodological constraints of the other classifiers, possibly allowing more accurate classification. Our study further demonstrates that the implementation of transfer learning is useful to overcome the issue that large datasets are needed to successfully train the CNN.

Our healthy subjects were younger than the patients. This is a possible limitation of our study, as there may be effects of age on normal walking which might influence classification to some degree. In a recent study, the lower-limb intersegmental coordination during walking was assessed kinematically and weak associations between age and kinematic parameters have indeed been found in a comparably large dataset of over 80 healthy participants: walking speed and range of motion in the ankle (55) were different for older and younger subjects. However, the magnitude of these effects was small and had not been found in another study, where no systematic kinematic differences during walking of healthy subjects between 15 and 70 years of age were detected (56). The impact of these differences on EMG patterns was not reported by these authors, but muscle activation patterns have been found to be similar in older and younger healthy subjects in another study (57), while being different for orthopedic patients and controls. Therefore, we suspect that the impact of the disorders (orthopedic or neurological) on gait patterns are comparably larger than the impact of age. More importantly, the finding that our classification was able to distinguish between two sets of gait disorders apart from differentiating between healthy controls and patients constitutes strong evidence that the signal patterns driving the classification were mainly not based on age effects.

In summary, we found that a CNN can be trained for automatic differentiation of gait disorders and healthy subjects based on EMG data allowing classification accuracy of 80 to 90% even in small sample sizes when applying transfer learning. To further improve on the method we will need to build a larger normative database containing subjects with different and more specified gait disorders, perhaps at different stages of their disease and different degrees of symptom load, which then can be used to train ever more precise artificial neural networks and classifiers. These are supposed to be able to identify disorders on an aetiopathological level instead into the broader classes of phenotypic gait disorders as demonstrated here, although mixed pathologies may constitute a major obstacle which may be difficult to assess. If these approaches prove to be successful objective classifications of walking abnormalities, the implementation of EMG in a screening assessment may help improving the diagnostic accuracy and hopefully the treatment during clinical practice. Although the approach appears feasible, the magnitude of benefit resulting from adding our or a similar classifier to the clinical diagnosis remains speculative. As the accuracy may lie above the general clinical accuracy some improvement could be inferred although the magnitude of improvement might well depend on the pretest probability of wrongly classifying a given patient. In particular, a gait disorder ambiguous even to the expert may remain ambiguous if it is classified using a classification system that is trained on unambiguous pathological features producing clearly distinguishable signals. This uncertainty within the gold standard may limit the accuracy and benefit of our and of similar classifiers substantially. This limitation may only eventually be overcome if a classifier's accuracy and its additional benefits are associated with histopathological (e.g., post mortem) markers.

## Data Availability Statement

The raw data supporting the conclusions of this article will be made available by the authors, without undue reservation.

## Ethics Statement

The studies involving human participants were reviewed and approved by Ethical Committee at the Medical Faculty, Leipzig University. The patients/participants provided their written informed consent to participate in this study.

## Author Contributions

CF: conception, data acquisition, data analysis, and writing of the manuscript. JA: conception, data analysis, and writing of the manuscript. NZ: conception, data acquisition, and data analysis. TW and MB: data analysis and writing of the manuscript. JC: conception, fund raising, data analysis, and writing of the manuscript. All authors approved the final version of the manuscript.

## Conflict of Interest

The authors declare that the research was conducted in the absence of any commercial or financial relationships that could be construed as a potential conflict of interest.
