# Supplementary material for: Evaluation of Three Machine Learning Algorithms for the Automatic Classification of EMG Patterns in Gait Disorders
Source: Front Neurol. 2021 May 21;12:666458. doi: 10.3389/fneur.2021.666458 (PMC8175858; doi:10.3389/fneur.2021.666458)
Supplement: Supplementary file 1 [file Data_Sheet_1.PDF]

Supplementary Material

EMG features

We used CNN, SVM and KNN to classify EMG data recorded from patients with gait disorders and healthy persons during walking with respect to the gait pathology present. For SVM and KNN we extracted 7 features (Phinyomark *et al.*, 2012) from EMG signals in time-domain and frequency-domain (after Fast Fourier Transformation) as defined in supplementary table 1.

Feature	Formula
Integrated EMG (IEMG)	$IEMG = \sum_{n=1}^{N} \|x_n\|$
Simple Square Integral (SSI)	$SSI = \sum_{n=1}^{N} \|x_n\|^2$
Variance (VAR)	$VAR = \frac{1}{N-1} \sum_{n=1}^{N} x_n^2$
Root Mean Square (RMS)	$RMS = \sqrt{\frac{1}{N} \sum_{n=1}^{N} x_n^2}$
Area of Power	$Area\ of\ Power = \sum_{j=1}^{M} ({P_j}/{M})^2$
Spectral Moment (SM1)	$SM1 = \sum_{j=1}^{M} P_j f_j$
Peak Frequency (PKF)	$PKF = \max(P_j), j = 1, \dots, M$

Supplementary table 1: Features used in SVM and KNN. N … number of data points of the EMG signal, x_n … nth data point, P_j … absolute value at frequency bin j after Fast Fourier Transformation of the EMG signal, f_j … value of frequency corresponding to frequency bin j, M … total number of frequency bins

SVM and KNN hyperparameters

We used a grid search approach to find suitable hyperparameters for training the SVM and KNN classifiers. For the SVM classifier, we need to optimize two hyperparameters C and Gamma, which were tested a the following values: C = [0.01,0.03,0.1,0.3,1,3,10,30], Gamma =[0.01,0.03,0.1,0.3,1,3,10,30]. For KNN we evaluated the classifier performance at values K = [1,2,3,…,30]. The best results were achieved using the parameters are reported in supplementary table 2. The table shows the results of the grid search optimization of the classifier hyperparameters when excluding a given participant from the validation and training set (the data from the left-out participant would then consitute the test set) as described in the Methods section..

	Healthy vs. patients			Healthy vs. hypokinetic vs. ataxic		
**Left-out participant**	C	Gamma	K	C	Gamma	K
1	3	0.01	29	30	10	2
2	0.01	0.01	4	1	1	2
3	30	0.01	15	3	3	2
4	0.01	0.01	2	3	10	4
5	0.01	0.01	2	1	3	5
6	0.01	0.01	30	1	3	23
7	0.01	0.01	1	30	10	2
8	0.01	0.01	17	10	10	1
9	3	0.01	1	30	10	5
10	30	0.01	28	1	10	10
11	0.01	0.01	27	3	10	20
12	0.01	0.01	17	3	3	17
13	0.01	0.01	29	1	10	2
14	0.01	0.01	27	10	10	5
15	1	0.01	2	1	1	1
16	0.01	0.01	2	3	10	28
17	0.01	0.01	1	1	30	1
18	3	0.01	2	30	10	3
19	0.01	0.01	1	30	10	2
20	10	0.01	7	1	10	2
21	0.01	0.01	1	10	30	19
22	3	0.01	27	3	3	29
23	0.01	0.01	2	10	30	1
24	0.01	0.01	19	30	10	2
25	30	0.01	23	30	10	1
26	3	0.01	4	10	10	17
27	30	0.01	4	1	1	1
28	3	0.01	2	0.3	3	1
29	0.01	0.01	25	30	10	13
30	0.01	0.01	29	10	10	1
31	0.01	0.01	29	1	3	18
32	0.01	0.01	2	30	10	3
33	10	0.01	1	30	10	27
34	0.01	0.01	1	1	10	5
35	0.01	0.01	29	10	30	2
36	0.01	0.01	2	3	10	1
37	0.01	0.01	2	0.1	10	13

Supplementary table 2: Optimized hyperparameters for SVM and KNN across subjects in both classification problems.

References

Phinyomark A, Phukpattaranont P, Limsakul C. Feature reduction and selection for EMG signal classification. Expert Systems with Applications 2012; 39: 7420–7431.
